# Detecting complexes from edge-weighted PPI networks via genes expression analysis

**DOI:** 10.1186/s12918-018-0565-y

**Published:** 2018-04-24

**Authors:** Zehua Zhang, Jian Song, Jijun Tang, Xinying Xu, Fei Guo

**Affiliations:** 1 School of Computer Science and Technology, Tianjin University, Tianjin, People’s Republic of China; 2 Tianjin University Institute of Computational Biology, Tianjin, People’s Republic of China; 3 School of Chemical Engineering and Technology, Tianjin University, Tianjin, People’s Republic of China; 4 Department of Computer Science and Engineering, University of South Carolina, Columbia, USA; 5 School of Information Engineering, Taiyuan University of Technology, Taiyuan, People’s Republic of China

**Keywords:** Complex detection, PPI networks, Edge-weighting scheme, Graph information, Gene expression analysis

## Abstract

**Background:**

Identifying complexes from PPI networks has become a key problem to elucidate protein functions and identify signal and biological processes in a cell. Proteins binding as complexes are important roles of life activity. Accurate determination of complexes in PPI networks is crucial for understanding principles of cellular organization.

**Results:**

We propose a novel method to identify complexes on PPI networks, based on different co-expression information. First, we use Markov Cluster Algorithm with an edge-weighting scheme to calculate complexes on PPI networks. Then, we propose some significant features, such as graph information and gene expression analysis, to filter and modify complexes predicted by Markov Cluster Algorithm. To evaluate our method, we test on two experimental yeast PPI networks.

**Conclusions:**

On DIP network, our method has Precision and F-Measure values of 0.6004 and 0.5528. On MIPS network, our method has F-Measure and *S*_*n*_ values of 0.3774 and 0.3453. Comparing to existing methods, our method improves Precision value by at least 0.1752, F-Measure value by at least 0.0448, *S*_*n*_ value by at least 0.0771. Experiments show that our method achieves better results than some state-of-the-art methods for identifying complexes on PPI networks, with the prediction quality improved in terms of evaluation criteria.

## Background

Detecting of protein complexes from PPI networks is a key problem to elucidate protein functions and identify biochemical, signal and biological processes in a cell. Like other biological molecules, most proteins do not work in isolation; they cooperate with other proteins to perform a particular biological function. These complexes are molecular aggregations of two or more proteins assembled by PPIs [1]. Accurate determination of complexes in PPI networks is crucial for understanding principles of cellular organization.

In past several years, a large number of technologies have been developed for the large-scale analysis of complex detection from PPI networks [2–13]. Heuristic-based algorithms find dense network regions by searching heuristically for potential cluster regions using an iterative greedy seed and extend strategy, one of the seminal efforts is MCODE [14] and proposed a density-based clustering approach to detect complexes, which picked vertices with large weights as initial clusters and further augmented them to detect dense connective clusters. Similar to MCODE, Altaf-UI-Amin proposed an algorithm called DPClus [15] with good accuracy. Li [16] proposed IPCA, it searches for subgraphs having small diameter and whose cluster property is above the interaction probability threshold. And *RestrictedNeighborhoodSearch* *Clustering* (*RNSC*) algorithm [17] deploys a cost-based partitioning algorithm. The *ClusterONE* method [18], detects overlapping clusters in a PPI network using a greedy seed and extend heuristic, an advantage of *ClusterONE* is the ability to not just find overlapping clusters, but also clusters that may be contained in another cluster.

One of the most widely used graph clustering algorithm is Markov Cluster Algorithm (MCL) [19], which is a fast and robust method, which simulate random walk in the graph to cluster. Lots of studies indicated that MCL can tolerate more noises than other clustring algorithms on PPI networks [8]. Algorithms such as R-MCL [20], SR-MCL [21], MCL-CA [22] and RRW [23] were proposed to overcome further weaknesses of MCL. However, SR-MCL still predicted too many complexes, and RRW predicted complexes of a particular size. On the basis of these limitations, we design a novel edge-weighting MCL method to detect complexes on PPI networks, which can effectively improve accuracy of clustering results. Then, there are some classic clustering algorithms that can be used on the *ClustEval* framework [24], such as DBSCAN [25], Spectral Clustering [26], Transitivity Clustering [27], fanny [28], but our study is not a complete clustering problem, classical clustering algorithm need combining with the post-processing or some improvements.

Complete enumeration algorithms aim to enumerate all possible subgraphs in G with density exceeding a specified threshold. Spirin and Mirny [29] proposed three techniques for detecting protein complexes and functional modules from PPI networks. The first approach finds cliques as modules by complete enumeration. The second approach leverages the notion of super-paramagnetic clustering (SPC), which assigns to each vertex a spin with several states. Lastly, they proposed a Monte Carlo optimization-based technique (MC) where finding highly connected set of vertices is formulated as an optimization problem. The CFinder method [30] identifies a set of k-clique modules in a PPI network where k-cliques correspond to k node complete subgraphs of G with a maximum density of 1. It is based on a deterministic approach called the Clique Percolation Method (CPM) [31], which generates overlapping clusters by finding k-clique percolation communities. Then, Cui [32] showed on the yeast PPI network that near-cliques may reveal better quality functional modules compared to overlapping cliques. The *Clustering*−*basedonMaximal* *Cliques* (*CMC*) [33] method generated maximal cliques from a weighted PPI network and combined or removed them, considering to connectivity and overlapping rate. A common theme among complete enumeration algorithms is exhaustive search. While such search enables identification of all relevant modules within a PPI network, it is computationally expensive. Therefore, their applications are limited to relatively small PPI networks.

Leung [34] developed a core-attachment approach for identifying complexes from PPI networks of single species and studying the organization of complexes. Ulitsky and Shamir [10] reformulated the problem of finding modules with high confidence connectivity as finding subgraphs to satisfy a weight threshold of their minimum cut. Shi [11] proposed a neural network-based semi-supervised learning method, which leverages proteomic features of subgraphs in a weighted PPI network with their topological features to generate complexes. Macropol [23] proposed a protein complex prediction algorithm, named by RRW, which constructed a cluster of proteins according to the stationary probability of a random walk. Maruyama [35] extended the RRW by introducing a random walk via restarts with a cluster of proteins, each of which is weighted by the sum of strengths for directly physical interactions. Also, Maruyama proposed a novel method based on random walks, Naive Bayes classifiers, and sampling methods [36–40].

Above methods only focus on static PPI networks. In reality, PPI networks in a cell are not static but dynamic [41–43]. The dynamic PPI network can be changing over time, environments and different stages of cell cycles [44, 45]. Lots of methods used dynamic PPI networks to predict complexes accurately [46, 47]. Li [12] proposed a new DPC algorithm to identify complexes based on gene expression profiles and PPI networks, based on static expressed core in all molecular cycles and short-lived dynamic attachments. Also, Luo [13] proposed a DCA method to identify more accurate protein complexes in dynamic PPI networks. Srihari [48] incorporated time in the form of cell-cycle phases into the analysis of complexes from PPI networks and studied the temporal phenomena of complex assembly and disassembly across phases.

Existing methods constructed PPI networks, based on gene expression variance of each protein [49, 50]. Segal [7] introduced a unified probabilistic model to detect functional modules from gene expression, based on the assumption that genes in the same pathway display similar expression profiles and products of genes work together to accomplish certain task. Maraziotis [9] proposed a DMSP algorithm finding functional modules by integrating gene expression and PPI data. In general, if a protein is at active time point, the expression level of corresponding gene is at the peak point. Some researchers use the dynamic information from gene expression data to construct time-evolving dynamic protein interaction networks, which divided proteins into active and inactive and combined active proteins at the same time to form a new network [12, 31, 42, 43, 49, 50]. We design a new co-expression analysis method to measure each protein complex, based on differential co-expression information. Different proteins in the same complex have similar trend on gene expression intervals.

We propose a novel method to identify complexes on PPI networks. First, we design an edge-weighting MCL method to calculate complexes on PPI networks. Second, we propose a novel co-expression analysis method to evaluate predicted complexes, based on differential co-expression information. To evaluate our method, we test on two experimental yeast PPI networks. On DIP network, our method has Precision and F-Measure values of 0.6004 and 0.5528. On MIPS network, our method has F-Measure values of 0.3774. Comparing to existing methods, our method improves Precision value by at least 0.1752, F-Measure value by at least 0.0448, *S*_*n*_ value by at least 0.0771.

## Methods

**Fig. 1:**
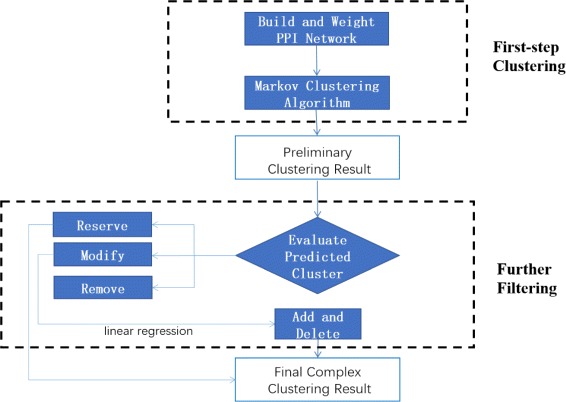
The overall process of our method and analysis pipeline to detect complexes from PPI network

We propose a novel method to identify protein complexes on PPI networks. First, we use Markov Cluster Algorithm with an edge-weighting scheme to calculate complexes on PPI networks. Second, we design a novel co-expression analysis method to measure each protein complex, based on differential co-expression information. Figure 1 shows the overall process of our method and the analysis pipeline to detect complexes from PPI network.

### Edge-weighting scheme

A PPI network is formulated as an undirected graph *G*=(*V*,*E*), where *v*_*i*_∈*V* represents a protein and (*v*_*i*_,*v*_*j*_)∈*E* denotes that protein *v*_*i*_ interacts with protein *v*_*j*_.

Given a graph *G*, *N*(*v*_*i*_) denotes all neighbors of *v*_*i*_ in the PPI network. Let *A* be a |*V*|×|*V*| adjacency matrix, and *A*(*i*,*j*) denotes the confidence weight of edge (*v*_*i*_,*v*_*j*_), defined as follows.

$$A(i,j)=\left\{ \begin{array}{lcl} \frac{|N(v_{i}) \cap N(v_{j})|}{|N(v_{i}) \cup N(v_{j})|} & & {if\ (v_{i},v_{j}) \in E}\\ \max_{k \ne j} \{A(i,k)\} & & {if\ v_{i}=v_{j}}\\ 0 & & else \end{array} \right. $$

where |*N*(*v*_*i*_)∩*N*(*v*_*j*_)| is the intersection of neighbors between *N*(*v*_*i*_) and *N*(*v*_*j*_), and |*N*(*v*_*i*_)∪*N*(*v*_*j*_)| is the union of neighbors between *N*(*v*_*i*_) and *N*(*v*_*j*_).

Since two vertices having a larger proportion of common neighbors, one vertex can move to another vertex with great probability. A canonical flow matrix *M* indicates the probability of transitions via a random walk, and *M*(*i*,*j*) represents the probability of a transition from *v*_*i*_ to *v*_*j*_, defined as follows.

$$M(i,j)=\frac{A(i,j)}{\sum_{k=1}^{n}A(k,j)} $$

where *n* is the number of all vertices in the graph, and each column of *M* sum up to 1.

### Markov cluster algorithm

Markov Cluster Algorithm proposed by Stijn van Dongen [19], is an iterative process of applying two operations, namely Expand and Inflate. These two operations are alternately applied to an initial stochastic matrix *M*, iterating until convergence. In addition, Prune is performed at the end of each iteration, in order to remove entries with very small values.

#### Expand and inflate

The operation of Expand is simply expressed as *M*_*exp*_=*M*×*M*. We calculate *M*_*exp*_ on the basis of *M*, and then assign the obtained matrix *M*_*exp*_ to *M*.

The operation of Inflate raises each entry in *M* using parameter *r*, and re-normalizes elements in each column that sum up to 1. Then, we assign the obtained matrix *M*_*inf*_ to *M*. The operation of Inflate, named by *M*_*inf*_(*i*,*j*), is expressed as follows.

$$M_{inf}(i,j) = \frac{M(i,j)^{r}}{\sum_{k=1}^{n} M(k,j)^{r}} $$

where *n* is the number of all vertices in the graph, and *r* is the parameter of Inflate, by default *r*=2,.

#### Prune

In the iterative process, there are some entries with very small values, and let these entries to be zero. This operation can make convergence faster, and keep the key part of aggregation information.

We use *L*_*j*_={*k*|*M*(*k*,*j*)>0} to represent a collection of vertices in column *j* with values greater than zero; in other words, it is a collection of vertices that flow to vertex *v*_*j*_. We calculate the average value of all elements in *L*_*j*_ as follows.

$$avg(j) = \frac{\sum_{k=1}^{|L_{j}|} M(L_{j}(k),j)}{|L_{j}|} $$

And also, we calculate the threshold to filter entries with small values in column *j* of *M* as follows.

$$thd(j) = avg(j) - w \times \frac{\sum_{k=1}^{|L_{j}|} (M(L_{j}(k),j) - avg(j))^{2}}{|L_{j}|} $$

where *w* is a parameter to adjust the threshold value, by default *w*=1.

We remove entries with very small values less than *thd*(*j*) in column *j* of *M*, filled with zero. After the operation, *M* must be re-normalized, and elements in each column of *M* sum up to 1.

#### Cluster

After lots of iterations, we find that most vertices flow to one vertex, and there exists one non-zero entry per column in the flow matrix *M*. We assign all vertices flowing to the same vertex as belonging to one cluster.

### Feature analysis

We propose some significant features, such as graph information and gene expression, to filter and modify complexes predicted by Markov Cluster Algorithm.

#### Connection

The direct connection (edge) in the PPI network, denotes that one protein interacts with another protein. We not only use the interaction information, but also consider indirect connection with a *n*-length shortest path as *n*-connection.

If there exists a *n*-connection between *v*_*i*_ and *v*_*j*_, we can define *Connect*(*v*_*i*_,*v*_*j*_,*n*)=1; else, *Connect*(*v*_*i*_,*v*_*j*_,*n*)=0. Moreover, *PathNum*(*v*_*i*_,*v*_*j*_,*n*) denotes the total number of *n*-length shortest paths from *v*_*m*_ to *v*_*n*_.

Given a protein *v*_*k*_ and a complex *C*, we calculate the ratio of *n*-connection proteins in *C* from *v*_*k*_, defined as follows.

$$ConnectRatio(v_{k},C,n) = \frac{\sum_{i=1}^{|C|} Connect(v_{k},v_{i},n)}{|C|} $$

Also, we calculate the ratio of total *n*-length shortest paths for *n*-connection proteins in *C* from *v*_*k*_, defined as follows.

$$PathRatio(v_{k},C,n) = \frac{\sum_{i=1}^{|C|} PathNum(v_{k},v_{i},n)}{|C|} $$

Here, we calculate these features of 2-connection and 3-connection.

#### Density

We can use the density of complex *C* to describe intensive degree of *n*-connection proteins, defined as follows.

$$Den(C,n) = \frac{\sum_{v_{i},v_{j} \in C}Connect(v_{i},v_{j},n)}{|C|\times|C|} $$

When a protein *v*_*k*_ is added into complex *C*, a new complex *C*^′^ can be formed. We calculate the density difference between these two complexes, as follows.

$$DenDiff(v_{k},C,n) = Den(C') - Den(C) $$

where *C*^′^={*C*,*v*_*k*_}.

Here, we also calculate these features of 2-connection and 3-connection.

#### Co-expression

Gene expression data could reflect features of proteins under various conditions in a biological process [43, 51]. It is the numerical expression value of one protein within the time period. For a protein, the fluctuation range of its expression value is not the same. We normalize each value to compare the similarity of expression intervals of proteins, as follows.

$$T_{i}'(l) = \frac{T_{i}(l)}{\max_{l}\{T_{i}(l)\}} $$

where *T*_*i*_(*l*) represents the expression value of protein *v*_*i*_ at the time point *l*.

**Fig. 2:**
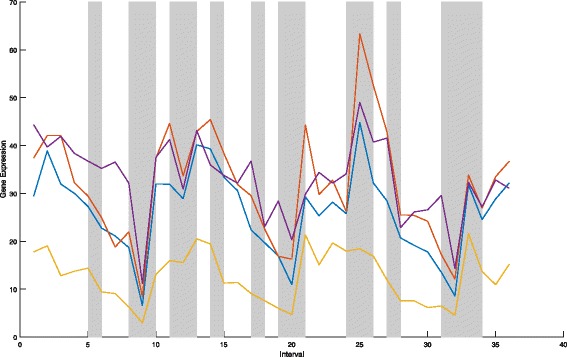
The gene expression data of *eIF*3 complex on 36 intervals

**Fig. 3:**
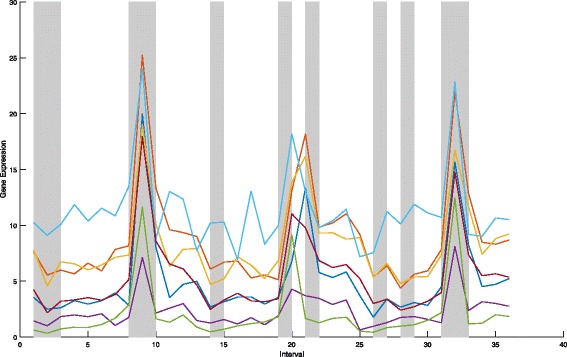
The gene expression data of Succinate Dehydrogenase complex (complex II) on 36 intervals

On 36 intervals,Fig. 2 shows the gene expression data for *eIF*3 complex, and Fig. 3 shows the gene expression data for Succinate Dehydrogenase complex (complex II). We find that six proteins in one complex tend to have similar tendency of expression values at the fixed time interval (indicated as gray-shadowed intervals).

Two proteins have a similar degree of expression at the same time interval leading to a high co-expression value. If the gene expression data of proteins are not similar, their co-expression value is low. Therefore, we calculate the co-expression value *E*_*co*_(*v*_*i*_,*v*_*j*_) of proteins *v*_*i*_ and *v*_*j*_, defined as follows.

$$E_{co}(v_{i},v_{j}) = \sum_{l=1}^{m} \ln \frac{T_{i}'(l) + T_{j}'(l)}{|T_{i}'(l) - T_{j}'(l)|} $$

where *m* is the number of expression intervals.

For a complex *C*, we can measure the co-expression value, as follows.

$$E_{co}(C) = \frac{\sum_{v_{i},v_{j} \in C} E_{co}(v_{i},v_{j})}{|C|\times|C|} $$

And, the co-expression value between one protein *v*_*k*_ and a complex *C* is defined as follows.

$$E_{co}(v_{k},C) = \frac{\sum_{i=1}^{|C|}E_{co}(v_{k},v_{i})}{|C|} $$

We calculate average co-expression value of all pairs of proteins in the PPI network, defined as *E*_*co*_(*avg*). If *E*_*co*_(*v*_*i*_,*v*_*j*_)>*E*_*co*_(*avg*), we set *Co*(*v*_*i*_,*v*_*j*_)=1, else, *Co*(*v*_*i*_,*v*_*j*_)=0.

We calculate the ratio of co-expression protein pairs in a given complex *C*, as follows.

$$CoRatio(C) = \frac{\sum_{v_{i},v_{j} \in C} Co(v_{i},v_{j})}{|C| \times |C|} $$

When a protein *v*_*k*_ is added into complex *C*, a new complex *C*^′^ can be formed. We calculate the co-expression difference between these two complexes, as follows.

$$CoDiff(v_{k},C) = E_{co}(C') - E_{co}(C) $$

where *C*^′^={*C*,*v*_*k*_}.

For protein *v*_*k*_, we calculate the number of co-expression proteins in a complex *C*, as follows.

$$CoProNum(v_{k},C) = \sum_{i=1}^{|C|} Co(v_{k},v_{i}) $$

Also, we calculate the ratio of co-expression proteins for protein *v*_*k*_ in a complex *C*, as follows.

$$CoProRatio(v_{k},C) = \frac{CoProNum(v_{k},C)}{|C|}. $$

### Complex detection

We set two thresholds, a lower bound and a higher bound for each of three features, to filtering predicted complexes: *Den*(*C*,*n*), *E*_*co*_(*C*), *Cotadio*(*C*). We reserve complexes with high qualities, discard complexes with low values, and modify median complexes. Algorithm 1 shows the overall algorithm of filtering method.



We use a linear function of seven features to determine how to modify a given complex: *ConnectRatio*(*v*_*k*_,*C*,*n*), *PathRatio*(*v*_*k*_,*C*,*n*), *DenDiff*(*v*_*k*_,*C*,*n*), *E*_*co*_(*v*_*k*_,*C*), *CoDiff*(*v*_*k*_,*C*), *CoProNum*(*v*_*k*_,*C*), *CoProRatio*(*v*_*k*_,*C*). We delete proteins in a complex with low qualities, and add neighbor proteins with high values into the complex. Algorithm 2 shows the overall algorithm of modifying method.



## Results and discussion

Experiments show that our method achieves better results than some state-of-the-art methods for identifying protein complexes on PPI networks, with the prediction quality improved in terms of many evaluation criteria.

### Data set

Our method is applied on two experimental yeast PPI networks. One is retrieved from the Database of Interacting Proteins (DIP) [52], which was used in COACH [34]. Another is downloaded from Munich Information Center for Protein Sequences (MIPS) database [53]. We remove self-connecting interactions and repeated interactions. The DIP network includes 4930 yeast proteins and 17,201 interactions, and the MIPS network contains 12,319 interactions among 4546 yeast proteins.

All predicted complexes are compared with the benchmark data, referred to as *CYC*2008 [54]. There are 408 manually annotated complexes, which are considered as the gold standard data.

We analyze gene expression data *GSE*3431 [55] downloaded from Gene Expression Omnibus (GEO), entitled as logic of the yeast metabolic cycle. This data set includes 6,777 gene products that cover more than 95% proteins in PPI networks.

### Assessment

At present, there are two popular measurements for evaluating the performance of complexes detection method, from many literatures [14, 56].

#### Sensitivity, Positive Predictive Value, Accuracy

In addition, Sensitivity (*S*_*n*_), Positive Predictive Value (*PPV*) and geometric Accuracy (*Acc*) have recently been proposed to evaluate the quality of protein complex prediction [56]. Give *n* benchmark complexes and *m* predicted clusters, let as *T*_*i*,*j*_ denote the number of common proteins between the *i*-th benchmark complex and the *j*-th predicted cluster. Then, *S*_*n*_, *PPV* and *Acc* are defined as follows.

$$\begin{array}{*{20}l} S_{n} &= \frac{\sum_{i=1}^{n} \max_{j}\{T_{i,j}\}}{\sum_{i=1}^{n}|C_{i}|}\\ PPV &= \frac{\sum_{j=1}^{m} \max_{i}\{T_{i,j}\}}{\sum_{i=1}^{n} \sum_{j=1}^{m} T_{i,j}}\\ Acc &= \sqrt{S_{n} \times PPV} \end{array} $$

Generally, *S*_*n*_ indicates that predicted complexes have a good coverage of proteins in benchmark complexes, and *PPV* indicates that predicted complexes are likely to be true positive. The geometric accuracy (*Acc*) indicates the tradeoff between *S*_*n*_ and *PPV*. It is obtained by computing the geometrical mean of them.

### Precision, Recall, F-measure

The overlapping score *O*(*C*_*p*_,*C*_*b*_) is used to assess how effectively a predicted complex *C*_*p*_ matches a benchmark complex *C*_*b*_ [14], defined as follows.

$$O(C_{p}, C_{b}) = \frac{|C_{p} \cap C_{b}|^{2}}{|C_{p}| \times |C_{b}|} $$

where |*C*_*p*_| is the number of proteins in the predicted complex, and |*C*_*b*_| is the number of proteins in the benchmark complex. If a predicted complex *C*_*p*_ that has no common proteins with a benchmark complex *C*_*b*_, then *O*(*C*_*p*_,*C*_*b*_)=0.

Usually, a predicted complex and a benchmark complex are considered as a match if their overlapping score is no less than a threshold value [14]. Let *P* be the set of complexes predicted by computational methods and *B* be the set of benchmark complexes in the PPI network. Then, the number of complexes in *P* at least matching one real complex is denoted by *N*_*cp*_=|{*C*_*p*_|*C*_*p*_∈*P*,∃*C*_*b*_∈*B*,*O*(*C*_*p*_,*C*_*b*_)≥*ω*}|, while the counterpart number in *B* can be denoted by *N*_*cb*_=|{*C*_*b*_|*C*_*b*_∈*B*,∃*C*_*p*_∈*P*,*O*(*C*_*p*_,*C*_*b*_)≥*ω*}|, by default *ω*=0.2.

Based on above definitions of *N*_*cp*_ and *N*_*cb*_, Precision and Recall can be defined as follows.

$$\begin{array}{*{20}l} Precision &= \frac{N_{cp}}{| P |}\\ Recall &= \frac{N_{cb}}{| B |} \end{array} $$

And, F-measure is their harmonic mean, defined as follows.

$$F-Measure = \frac{2 \times Precision \times Recall} {Precision + Recall} $$

### Filtering threshold

We consider three features *Vector*(*C*) to filtering predicted complexes. Based on co-expression value as *Eco*(*C*), *CoRatio*(*C*) as well as graph information as *Den*(*C*,*n*), the preliminary complex clustering result will be determined to be either reserved, removed or further modified. Two thresholds including a upper bond and a lower bond are set for each of the three features. The Tables 1, 2 and 3 show the extent of improvement of our result enhanced solely by each one of the three features respectively under distinctive thresholds. Both the gene expression and graph information are effective and make a contribution to a significantly improved result by 0.04 to 0.10 in Precision.

**Table 1:** Validity of our filtering threshold parameters (*max*,*min*) of *E*_*co*_(*C*)

*E*_*co*_(*C*)	|*complex*|	*N*_*cp*_	*N*_*cb*_	*S*_*n*_	PPV	Acc	Precision	Recall	F-Measure
(+*∞*,0)	1563	739	231	0.5464	0.4887	0.5167	0.4728	0.5662	0.5153
(+*∞*,50)	1466	717	225	0.5448	0.4885	0.5159	0.4891	0.5515	0.5184
(80,0)	1563	757	228	0.5641	0.4930	0.5273	0.4843	0.5588	0.5189
(80,40)	1540	754	227	0.5640	0.4929	0.5273	0.4896	0.5564	0.5209
(80,42)	1532	753	226	0.5640	0.4927	0.5272	0.4915	0.5539	0.5209
(80,44)	1519	749	226	0.5635	0.4924	0.5268	0.4931	0.5539	0.5217
(80,46)	1504	746	226	0.5630	0.4922	0.5264	0.4960	0.5539	0.5234
(80,48)	1487	740	223	0.5625	0.4926	0.5264	0.4976	0.5466	0.5210
(80,50)	1466	735	222	0.5625	0.4929	0.5266	0.5014	0.5441	0.5219
(80,52)	1434	728	220	0.5604	0.4937	0.5260	0.5077	0.5392	0.5230
(80,54)	1409	724	219	0.5599	0.4955	0.5267	0.5138	0.5368	0.5251
(80,56)	1375	713	216	0.5583	0.4967	0.5266	0.5185	0.5294	0.5239
(80,58)	1323	697	212	0.5542	0.4986	0.5256	0.5268	0.5196	0.5232
(80,60)	1265	669	206	0.5464	0.5015	0.5234	0.5289	0.5049	0.5166
(70,50)	1466	737	216	0.5688	0.4907	0.5283	0.5027	0.5294	0.5157
(75,50)	1466	735	215	0.5656	0.4916	0.5273	0.5014	0.5270	0.5138
(80,50)	1466	735	222	0.5625	0.4929	0.5266	0.5014	0.5441	0.5219
(85,50)	1466	727	224	0.5547	0.4922	0.5225	0.4959	0.5490	0.5211
(90,50)	1466	723	224	0.5531	0.4913	0.5212	0.4932	0.5490	0.5196
All	1563	756	213	0.5812	0.4871	0.5321	0.4836	0.5221	0.5021

**Table 2:** Validity of our filtering threshold parameters (*max*,*min*) of *CoRatio*(*C*)

*CoRatio*(*C*)	|*complex*|	*N*_*cp*_	*N*_*cb*_	*S*_*n*_	PPV	Acc	Precision	Recall	F-Measure
(0.60,0)	1409	730	213	0.5771	0.4907	0.5321	0.5181	0.5221	0.5201
(0.65,0)	1409	730	212	0.5755	0.4908	0.5315	0.5181	0.5196	0.5189
(0.70,0)	1409	735	215	0.5750	0.4911	0.5313	0.5216	0.5270	0.5243
(0.75,0)	1409	735	215	0.5740	0.4920	0.5314	0.5216	0.5270	0.5243
(0.80,0)	1409	731	213	0.5719	0.4930	0.5310	0.5188	0.5221	0.5204
(0.85,0)	1409	729	213	0.5677	0.4933	0.5292	0.5174	0.5221	0.5197
(0.90,0)	1409	727	211	0.5661	0.4936	0.5286	0.5160	0.5172	0.5166
(0.75,0.10)	1404	734	215	0.5740	0.4919	0.5313	0.5228	0.5270	0.5249
(0.75,0.15)	1404	734	215	0.5740	0.4919	0.5313	0.5228	0.5270	0.5249
(0.75,0.20)	1402	734	215	0.5740	0.4919	0.5313	0.5235	0.5270	0.5252
(0.75,0.25)	1399	733	214	0.5739	0.4918	0.5312	0.5239	0.5245	0.5242
(0.75,0.30)	1393	730	213	0.5729	0.4922	0.5310	0.5240	0.5221	0.5231
(0.75,0.35)	1368	718	211	0.5708	0.4919	0.5299	0.5249	0.5172	0.5210
(0.75,0.40)	1365	718	211	0.5708	0.4919	0.5299	0.5260	0.5172	0.5215
All	1563	756	213	0.5812	0.4871	0.5321	0.4836	0.5221	0.5021

**Table 3:** Validity of our filtering threshold parameters (*max*,*min*) of *Den*(*C*,2)

*Den*(*C*,2)	|*complex*|	*N*_*cp*_	*N*_*cb*_	*S*_*n*_	PPV	Acc	Precision	Recall	F-Measure
(0.14,0)	1409	736	214	0.5750	0.4956	0.5338	0.5224	0.5245	0.5234
(0.16,0)	1409	735	213	0.5734	0.4959	0.5333	0.5216	0.5221	0.5218
(0.18,0)	1409	741	218	0.5714	0.4959	0.5323	0.5259	0.5343	0.5301
(0.20,0)	1409	741	217	0.5708	0.4964	0.5323	0.5259	0.5319	0.5289
(0.22,0)	1409	738	217	0.5698	0.4962	0.5317	0.5238	0.5319	0.5278
(0.24,0)	1409	738	217	0.5698	0.4961	0.5317	0.5238	0.5319	0.5278
(0.26,0)	1409	734	217	0.5693	0.4963	0.5315	0.5209	0.5319	0.5263
(0.18,0.04)	1152	678	208	0.5406	0.5203	0.5304	0.5885	0.5098	0.5464
(0.18,0.05)	1128	663	207	0.5349	0.5251	0.5300	0.5878	0.5074	0.5446
(0.18,0.06)	1094	649	207	0.5328	0.5288	0.5308	0.5932	0.5074	0.5469
(0.18,0.07)	1079	640	205	0.5281	0.5308	0.5295	0.5931	0.5025	0.5440
(0.18,0.08)	1059	630	205	0.5250	0.5347	0.5298	0.5949	0.5025	0.5448
(0.18,0.09)	1034	613	204	0.5182	0.5371	0.5276	0.5928	0.5000	0.5425
(0.18,0.10)	1027	606	202	0.5099	0.5400	0.5247	0.5901	0.4951	0.5384
All	1563	756	213	0.5812	0.4871	0.5321	0.4836	0.5221	0.5021

In addition, the optimized parameter thresholds are obtained from the three tables.The minimum value of *E*_*co*_(*C*) can be set to 50; that is, our method removes complexes with low co-expression values, and *N*_*cb*_ and Recall decrease slightly, but Precision increases a lot. The maximum value of *E*_*co*_(*C*) can be set to 80; that is, our method reserves complexes with high co-expression values, and Precision and F-Measure increase slightly. Moreover, the threshold of *CoRatio*(*C*) can be set to (0.75,0.20), and the threshold of *Den*(*C*,*n*) can be set to (0.18,0.06).

### Modifying analysis

We use a linear function of seven features to evaluate the probability of a protein adding into a complex, as follows.

$$\begin{array}{*{20}l} L(v_{k},C) &= \sum_{i=1}^{7} w_{i} \times Feature_{i}(v_{k},C)\\ P(v_{k},C) &= \frac{1}{1 + e^{-L(v_{k},C)}} \end{array} $$

We discuss the effectiveness of our linear function, by using some randomly generated positive and negative samples to regression. First, we randomly select a protein in the network, and choose the size of generated complex. Then, we randomly choose another protein from the collection of neighbors. Repeat this step until producing a complex. Finally, we calculate the neighboring collection of this generated complex. If adding a neighboring protein makes new complex better than old one, we assign it is positive; else, it is negative. Similarly, we traverse all proteins in this complex. If deleting a internal protein makes new complex better than old one, we assign it is positive; else, it is negative. We use 1894 positive samples and 9309 negative samples to produce the optimal parameters, as *w*={0.01,0.02,0.01,0.24,0.36,0.03,0.33}. Gene Expression information as *CoProRatio*(*vk*,*C*),*CoDiff*(*vk*,*C*), *Eco*(*vk*,*C*) contribute the most.

#### Deleting

For *CYC*2008, we filter some complexes with less than three proteins, keeping 236 complexes with average size of 6.68 proteins. For each complex, we randomly put in a protein from PPI network to form a new complex. When randomly delete a protein, the probability of correct deleting is $P_{random} = \frac {1}{1 + 6.68} \approx 0.130$, but accuracy of our deleting method is 0.48. When randomly putting in two or three proteins, accuracy of our deleting method are 0.63 and 0.70, respectively. The analysis of deleting method is shown in Table 4.

**Table 4:** Validity of our deleting method

Errors	No. of Deleting	No. of Correct Deleting	Acc
1	1000	480	0.480
1	10000	4818	0.4818
2	1000	620	0.620
2	1000	635	0.635
2	10000	6367	0.6367
2	10000	6317	0.6317
3	10000	7056	0.7056
3	10000	7024	0.7024
3	10000	7065	0.7065

#### Adding

On DIP network, we generate 1507 complexes and use *P*(*v*_*k*_,*C*) for adding neighboring proteins. When randomly add a protein, the probability of correct adding is $P_{random} = \frac {6189}{105553} \approx 0.0586$. However, accuracy of our adding method is 0.0765 if *P*>0.5, and accuracy of our adding method is 0.3333 if *P*>0.8. The analysis of adding method is shown in Table 5.

**Table 5:** Validity of our adding method

	No. of Adding	No. of Correct Adding	Acc
*P*>0.5	54725	4189	0.0765
*P*>0.6	2127	368	0.1730
*P*>0.7	249	73	0.2932
*P*>0.8	78	26	0.3333
All	105553	6189	0.0586

### Comparison to existing methods

We compared the performance of our method with three existing methods, such as COACH, ClusterONE and MCL. COACH is a novel core-attachment method to detect complexes with two stages [34]. It detected cores of complexes and then added attachments into these cores to form biologically meaningful structures. ClusterONE is a method for detecting potentially overlapping complexes from the PPI data, clustering with overlapping neighborhood expansion [18]. MCL is a graph clustering algorithm based on stochastic flow simulation [19], which is effective in clustering biological networks. To evaluate our method, we test on two experimental yeast PPI networks.

On DIP network, results by our method and three existing methods are shown in Table 6. Our method has Precision and F-Measure values of 0.6004 and 0.5528. COACH achieves Precision and F-Measure values of 0.2896 and 0.3211, ClusterONE achieves Precision and F-Measure values of 0.4252 and 0.3675, and MCL achieves Precision and F-Measure values of 0.2353 and 0.2941. Comparing to existing methods, our method improves Precision value by at least 0.1752, and F-Measure value by at least 0.1853.

**Table 6:** Results by our method and three existing methods on DIP network

	|*P*|	*N*_*cp*_	*N*_*cb*_	*S*_*n*_	PPV	Acc	Precision	Recall	F-Measure
Our Method	1081	649	209	0.5313	0.5300	0.5306	0.6004	0.5123	0.5528
MCL	799	188	160	0.7776	0.2551	0.4454	0.2353	0.3922	0.2941
Coach	746	216	147	0.4245	0.5222	0.4708	0.2896	0.3603	0.3211
ClusterONE	341	145	132	0.3609	0.6701	0.4918	0.4252	0.3235	0.3675

On MIPS network, results by our method and three existing methods are shown in Table 7. Our method has F-Measure value of 0.3774. COACH achieves F-Measure values of 0.2497, ClusterONE achieves F-Measure values of 0.3326, and MCL achieves F-Measure values of 0.3158. Comparing to existing methods, our method improves F-Measure value by at least 0.0448, and also improves *S*_*n*_ value by at least 0.0771. Although our method did not achieve the best recall value, as we can see from the table, method with high recall values like DPClus and RRW, unavoidably have a particular poor precision, which indicates the high recall is based on counting into a overall large number of clusters and hence the precision is weakened. Comparatively, our method remains a relatively high recall value and achieves the best precision revealing the overall efficiency of our model.

**Table 7:** Results by our method and three existing methods on MIPS network

	|*P*|	*N*_*cp*_	*N*_*cb*_	*S*_*n*_	PPV	Acc	Precision	Recall	F-Measure
Our Method	866	354	143	0.3453	0.3766	0.3606	0.4088	0.3505	0.3774
MCL	658	273	104	0.2531	0.4050	0.3202	0.4149	0.2549	0.3158
Coach	489	135	93	0.2682	0.3797	0.3191	0.2760	0.2279	0.2497
ClusterONE	293	116	117	0.2521	0.6603	0.4080	0.3959	0.2794	0.3326

An available COACH system is downloaded from http://www.comp.nus.edu.sg/~lixl/, and a fast and free implementationof ClusterONE is available at http://www.paccanarolab.org/cluster-one/. Also, we compare our method to many other complex detection methods in Table 8, and results of these methods are from many literatures [10, 14, 30, 33].

**Table 8:** Results by our method and other complex detection methods

	Data Set	Precision	Recall	F-Measure
Our Method	DIP	0.6004	0.5123	0.5528
R-MCL	DIP	0.2923	0.3995	0.3376
SR-MCL	DIP	0.3281	0.4191	0.3680
Our Method	MIPS	0.409	0.351	0.3774
CMC	MIPS	0.339	0.346	0.3425
CFinder	MIPS	0.395	0.302	0.3423
DPClus	MIPS	0.204	0.531	0.2948
MCode	MIPS	0.330	0.241	0.2786
RRW	MIPS	0.193	0.517	0.2811

### Running time

Our experiments are conducted on a PC with Intel(R) Xeon(R) CPU E5-1620 of 3.7 GHz and 12.0 GB RAM. Here, we compare the running time of different methods on the PPI network with 4930 nodes and 17201 edges. Our method completes complex detection within 736 s, as shown in Table 9.

**Table 9:** Running time of different methods on the PPI network

	*Runtime*(*sec*)
Our Method	736
MCL	1924
Coach	221
ClusterONE	155

## Conclusions

We propose a novel method to identify complexes on PPI networks. First, we design an edge-weighting MCL method to calculate complexes on PPI networks. Second, we propose some significant features, such as graph information and gene expression, to filter and modify complexes predicted by Markov Cluster Algorithm.

Experiments show that our method achieves better results than some state-of-the-art methods for identifying complexes on PPI networks. To evaluate our method, we test on two experimental yeast PPI networks. On DIP network, our method has Precision and F-Measure values of 0.6004 and 0.5528, improves by at least 0.1752 and 0.1853. On MIPS network, our method has F-Measure value of 0.3774, improves by at least 0.0448.

## Abbreviations

CMC: Clustering?based on maximal cliques
CPM: Clique percolation method
Den: Density
DenDiff: Density difference
DIP: Database of interacting proteins
GEO: Gene expression omnibus
MC: Monte Carlo optimization-based technique
MCL: Markov cluster algorithm
MIPS: Munich information center for protein sequences database
PathNum: Path number
PPI: Protein protein interaction
RNSC: Restricted neighborhood search clustering
SPC: Super-paramagnetic clustering
